# Photoinhibition of *Streptococcus mutans* Biofilm-Induced Lesions in Human Dentin by Violet-Blue Light

**DOI:** 10.3390/dj7040113

**Published:** 2019-12-11

**Authors:** Grace Gomez Felix Gomez, Frank Lippert, Masatoshi Ando, Andrea F. Zandona, George J. Eckert, Richard L. Gregory

**Affiliations:** 1 Department of Biomedical Sciences and Comprehensive Care, Indiana University School of Dentistry, Indianapolis, IN 46202, USA; gfelixgo@iupui.edu; 2 Department of Cariology, Operative Dentistry and Dental Public Health, Indiana University School of Dentistry, Indianapolis, IN 46202, USA; flippert@iu.edu (F.L.); mando@iu.edu (M.A.); 3 Department of Comprehensive Care, Tufts School of Dental Medicine, Boston, MA 02111, USA; andrea.zandona@tufts.edu; 4 Department of Biostatistics, Indiana University, Indianapolis, IN 46202, USA

**Keywords:** violet-blue light, phototherapy, *Streptococcus mutans*, dental caries

## Abstract

This in vitro study determined the effectiveness of violet-blue light on *Streptococcus mutans* (UA159) biofilm induced dentinal lesions. Biofilm was formed on human dentin specimens in a 96-well microtiter plate and incubated for 13 h in the presence of tryptic soy broth (TSB) or TSB supplemented with 1% sucrose (TSBS). Violet-blue light (405 nm) from quantitative light-induced fluorescence (QLF^TM^) was used to irradiate the biofilm. Supernatant liquid was removed, and the biofilm was irradiated continuously with QLF for 5 min twice daily with an interval of 6 h for 5 d, except with one treatment on the final day. Colony forming units (CFU) of the treated biofilm, changes in fluorescence (∆F; QLF-Digital Biluminator^TM^), lesion depth (L), and integrated mineral loss (∆Z; both transverse microradiography) were quantified at the end of the fifth day. Statistical analysis used analysis of variance (ANOVA), testing at a 5% significance level. In the violet-blue light irradiated groups, there was a significant reduction (*p* < 0.05) of bacterial viability (CFU) of *S. mutans* with TSB and TSBS. Violet-blue light irradiation resulted in the reduction of ∆F and L of the dentinal surface with TSBS. These results indicate that violet-blue light has the capacity to reduce *S. mutans* cell numbers.

## 1. Introduction

Theories on the causation of dental caries are still evolving, but it is widely known as a chronic and infectious disease involving multiple factors with pandemic distribution [1,2,3]. Early dental caries is reversible and preventable. There are various methods to prevent dental caries by reducing, inhibiting, or eliminating caries-causing microorganisms. Alternative new approaches currently investigated vary from natural products to nanotechnological methods [4]. Among them, phototherapy with no exogenous photosensitizers or photodynamic therapy with exogenous photosensitizers are innovative approaches being studied to control oral biofilm [5,6,7,8,9,10,11,12].

Blue light within wavelengths of 400–500 nm has a delayed antibacterial activity on *S. mutans* biofilm by affecting their re-organization [11]. Feuerstein et al. demonstrated a combined activity of light and hydrogen peroxide on the growth of *S. mutans*. This study exclusively included a continuous 10 min of light treatment and showed a decrease of 3% in bacterial growth with blue light compared with the nontreated control [12,13]. Photodynamic therapies using external photosensitizers are more prevalent compared with phototherapy studies using only light as a source of treatment. Light therapy studies on oral bacteria without exogenous photosensitizers are limited and have been evolving in the past few years [12,13,14]. Our previous research demonstrated inhibitory effects of violet-blue light treatment on *S. mutans* growth and biofilm formation. The use of quantitative light-induced fluorescence (QLF) as a source of light with a peak wavelength at 405 nm to mediate bacterial destruction was developed in our previous study [15]. Metabolic activity of *S. mutans* biofilm with response to violet-blue light at 0, 2, and 6 h of re-incubation have been reported [16]. The effectiveness of violet-blue light on *S. mutans* biofilm grown on human enamel specimens has been studied previously [17].

*S. mutans* has different interactions with dentin and enamel. Dentin has more organic content and is less mineralized than enamel. Virulence of *S. mutans* is affected by its adherence to collagen in dentin causing cariogenicity [18]. Photodynamic therapies have been conducted previously with carious dentin [19,20,21,22,23]. The present study extends the application of an early caries detection device, QLF, to the field of treating *S. mutans* biofilm, the etiological factor of caries, with only violet-blue light. The effectiveness of violet-blue light at the most virulent timepoint of an *S. mutans* infection with the greatest adherence to dentin was studied. The aim of the present study was to test the inhibitory effects of violet-blue light on *S. mutans* biofilm induced dentinal lesions on human dentin specimens.

## 2. Materials and Methods

### 2.1. Study Design

**Figure 1:**
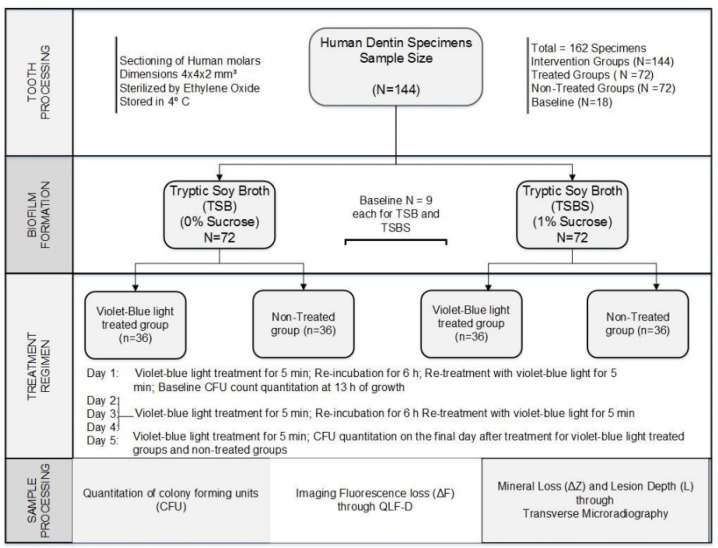
Flow chart of the study design of human dentin specimens. QLF, quantitative light-induced fluorescence.

A total of 162 human dentin specimens were used in this experiment. One hundred and forty-four (N = 144) specimens from the total available population were randomized into two groups with 72 specimens each, based on *S. mutans* biofilm grown with tryptic soy broth (TSB, Acumedia, Baltimore, MA, USA) or TSB supplemented with 1% sucrose (TSBS). The TSB or TSBS groups were further subdivided into 4 intervention groups with 36 specimens each with *S. mutans* biofilm grown with TSB with and without violet-blue light treatment and *S. mutans* biofilm grown with TSBS with and without violet-blue light treatment. The remaining 18 specimens were used for baseline measurements. *S. mutans* biofilm grown on the dentin was subjected to violet-blue light treatment for 5 min twice daily for 5 d with a 6 h re-incubation between treatments, except with one treatment on the final day. Specimens were analyzed on the final day of the treatment (Figure 1) [14,17].

### 2.2. Bacterial Strain and Culture Conditions

*S. mutans* (UA159, serotype c, ATCC 700610) from American Type Culture Collection (Rockville, MD, USA) stored at −80 °C in 20% glycerol was used in this study. The bacteria were cultured on mitis-salivarius sucrose bacitracin (MSSB, Anaerobe Systems, Morgan Hill, CA, USA) agar plates prior to use. *S. mutans* broth cultures were started by inoculating 5 mL of TSB with colonies from the MSSB plates and incubated for 24 h in a 5% CO_2_ incubator [24].

### 2.3. Light Source

The QLF (QLF-clin, Inspektor Research Systems BV, Amsterdam, The Netherlands) used in this study was an early caries detection light device with an excitation peak wavelength of 405 nm and a spectral range of 380 to 450 nm. Visible light with a spectral range from 380 to 700 nm is commonly used to inhibit or kill bacteria [9,25,26,27]. It is used clinically to detect changes in the mineral content of the tooth in a noninvasive manner, identify early lesions that will likely progress to cavitation, quantitatively measure the fluorescence, and assist in clinical decision-making. It employs a 35-watt xenon arc lamp and violet-blue light is filtered through a high pass band filter. The intensity of the violet-blue light from the QLF device used in this study was 13 mW/cm^2^ [28,29,30].

### 2.4. Selection of Tooth Specimens

Human teeth were collected after IUPUI/Clarian Institutional Review Board (IRB) approval (# NS0911-07) on 27 November 2009. One hundred and sixty-two specimens of extracted human molars without any cracks, fractures, or caries were selected. A Lap Craft L’il Trimmer^TM^ (Powell, OH, USA) was used to decoronate the crown portion of the teeth. The dentin portion from the coronal part of the teeth was obtained by cutting a circumferential section of the crowns. Dentin specimens with a dimension of 4 × 4 × 2 mm^3^ were cut using a Buehler Isomet saw (Buehler, Lake Bluff, IL, USA). The specimens were sequentially ground with 500, 1200, 2400, and 4000 grit sandpaper by mounting them on an acrylic block with a RotoPol-31/RotoForce-4 grinding machine (Struers, Cleveland, OH, USA). Each specimen was ground with the different sandpapers for 4 s and was reduced to a thickness of 2 mm. Flaws in the dentin specimens were evaluated through microscope at 20× magnification. Specimens with fractures, cracks, or fissures were discarded [31]. Clear nail varnish was coated on all sides and the bottom leaving approximately one-third of the top free of varnish for growth of the biofilm. Dentin specimens were rinsed, sonicated, and rinsed again for 3 min with deionized water (dH_2_O). The specimens were placed with moist cotton gauze, sealed in a whirl pak bag, and sterilized with ethylene oxide gas. They were stored at 4 °C until further use [32].

### 2.5. Quantification of Biofilm Formation

An overnight *S. mutans* TSB culture was diluted 1:100 with TSB or TSBS. Then, 10 µL of the diluted *S. mutans* culture was added to sterile 96-well microtiter plates (Fisher Scientific, Co., Newark, DE, USA) with 190 µL of TSB or TSBS in each well [33]. The bacteria were incubated with the dentin specimens (n = 72 in each TSB/TSBS group) for 13 h in a 5% CO_2_ incubator for them to reach the logarithmic phase of growth and produce biofilm. A gap of one well was placed in between each of the biofilm samples and two wells between the TSB and TSBS groups. Nine samples with and without sucrose were also simultaneously inoculated for baseline measurement of CFUs without QLF treatment at 13 h. Before irradiation, planktonic bacteria in the supernatant liquid were gently removed by aspirating from the side of the well in the microtiter plate. Immediately after aspiration, the wet biofilm was irradiated continuously for 5 min with violet-blue light. A 2 cm distance was kept between the light source and the bottom of microtiter plate. The instrument setup and the bottom of the plate was kept on a black background to prevent light scattering. Sterility of the biofilm was maintained by a clear seal mate (Excel Scientific Inc., Victorville, CA, USA), which was placed as a barrier between the light source and the well opening of the titer plate. The irradiated *S. mutans* biofilm was re-incubated in a 5% CO_2_ incubator for 6 h. The biofilm cells were treated again after 6 h for 5 min. The treated and the non-treated biofilm cells were re-incubated until the next treatment for 13 h. The procedure was repeated twice a day for 5 d, except on the final day, when only one treatment was provided followed by a 6 h re-incubation [17].

Biofilm was gently washed once with 0.9% saline to remove unattached and loosely adherent bacteria. Then, the dentin specimens were placed in mini-centrifuge tubes with 1 mL of saline. Sequentially, they were vortexed for 10 s, sonicated for 20 s in ice, and vortexed for 10 s again. Bacterial samples were serially diluted in saline and plated using a spiral plater (Spiral System^TM^, Cincinnati, OH, USA) on tryptic soy agar (TSA) plates. Enumeration of CFUs was calculated through an automated colony counter (Synbiosis Inc., Fredrick, MD, USA) after 48 h of incubation in 5% CO_2_ at 37 °C [15,17].

### 2.6. Quantitative Light-Induced Fluorescence

A QLF-D biluminator^TM^ 2 (Inspektor Research Systems BV, Amsterdam, The Netherlands) was used to acquire images of the dentin specimens used above for bacterial quantitation. A custom-made jig was prepared with silicone rubber (Oomo-30) where the dentin specimen will properly fit. Fluorescent images were acquired under dark environment through a ring of white and blue light emitting diodes (LED) around the lens of a single lens reflex (SLR) camera. C3 proprietary software (Inspektor Research Systems BV, Amsterdam, The Netherlands) with a 60-mm macro lens, shutter speed of 1/30 s, and an International Standards Organization (ISO) speed of 1600 with an aperture value of 8 was used to capture the fluorescent images. The images were stored digitally for further analysis for QLF-D parameters through QA2 analysis software (Inspektor Research Systems BV, Amsterdam, The Netherlands). The parameter ∆F represents fluorescence radiance loss (%), an indirect measure equivalent to lesion depth, ∆Q signifies a measure of ∆F (%) integrated over the area of lesion A_∆F_ (px^2^) resembling lesion volume (%px^2^), and ∆Fmax is the maximum amount of fluorescence loss (%) in radiance [34,35].

### 2.7. Transverse Microradiography

Before sectioning, the human dentin specimens were preserved with 0.01% thymol after a brief treatment with 70% ethyl alcohol. They were bonded and fixed to acrylic rods for sectioning. Sections with an approximate thickness of 180 µm were prepared using a hard tissue microtome. Plastic sheet wrapped ultra-resolution flat plate sized 5 × 5 × 2 mm^3^ (Microchrome Technology Inc., San Jose, CA, USA) was used to arrange the dentin specimens for imaging. A Transverse Microradiography Photonic Science Limited (TMR PSL) Imaging System (Thermo-Kevex PXS5-928WB-LV, Tube 48934) was calibrated for its absorption coefficient. An aluminum step wedge with 14 steps was used for calibration by placing it in the sample holder for an acceptable correlation of 0.99970. Acquisition of dentin images were obtained through an X-ray source with a voltage 45 kV and a similar current with 45 µA. The images were read through TMRD1 5.0.01 software and processed and analyzed by TMR2006 computer software v.3.0.0.18. Mineral loss (∆Z; assuming 50% v/v mineral content of sound dentin) and lesion depth (L; 47.5% mineral, i.e., 95% of the mineral content of sound dentin) were determined.

### 2.8. Statistical Methods

Sample size and power analysis were calculated through a statistical analysis system (SAS) statistical program (Cary, NC, USA). At a 5% significance level, 36 dentin specimens for each TSB or TSBS group would provide 80% power to detect a 25% difference in CFUs and a 10 µm difference in lesion depth. A logarithmic transformation was used for all analyses. ANOVAs with fixed effects for group (violet-blue light, no light, baseline), media (TSB, TSBS), and their interaction, as well as a random effect for experiment within each media, were used to compare the groups with data from the experiments combined into a single analysis. Each group-media combination was allowed to have a different variance.

## 3. Results

### 3.1. Photo Inhibitory Effect on Colony Forming Units

**Figure 2:**
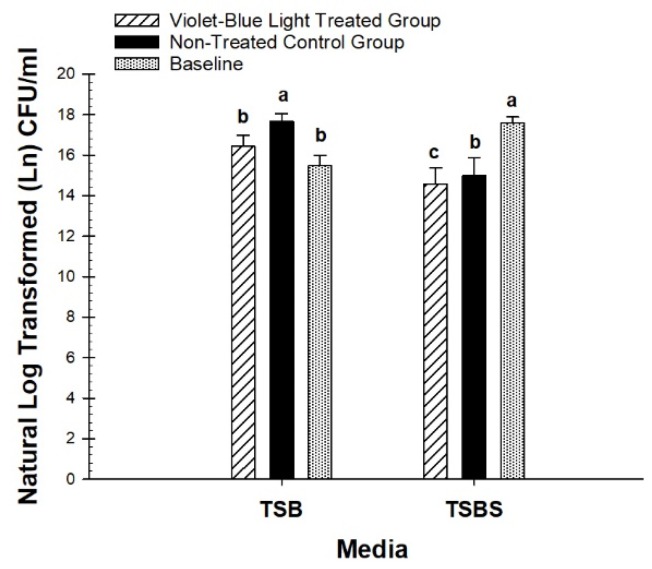
Comparison of baseline colony forming unit (CFU) between treated and non-treated groups. Different letters represent significant differences between groups within tryptic soy broth (TSB) and TSB supplemented with 1% sucrose (TSBS), respectively.

The quantitation of *S. mutans* biofilm was based on the comparison of violet-blue light-treated (n = 36) and non-treated groups (n = 36) at the end of the treatment period in human dentin specimens. At the end of the fifth day of treatment, there was a statistically significant (*p* = 0.0006) decrease of CFU in the treated groups with TSB-grown biofilm (n = 72). There was a 60.6 percent reduction of bacterial colonies without sucrose in the treated groups compared with the non-treated groups (Figure 2).

For TSBS-grown biofilm, there was also a significant decrease (*p* = 0.0337) in CFU in the violet-blue light-treated groups (n = 36). There was a 28.8% reduction of bacterial colonies in sucrose-grown groups (Figure 2). Violet-blue light treated TSB and TSBS groups were significantly decreased compared with the respective non-treated groups.

The violet-blue light-treated groups were not significantly different from the baseline for TSB (*p* = 0.07), however, non-treated groups were significantly higher than the baseline for TSB (*p* = 0.0001). CFU of the violet-blue light-treated groups and non-treated groups was significantly lower than the baseline for TSBS (*p* < 0.0001). TSB-grown biofilm was significantly higher than TSBS-grown biofilm for the violet-blue light treated (*p* = 0.0480) and non-treated (*p* = 0.0042) groups, however, there were no differences in the baseline CFU between TSB and TSBS (*p* = 0.06).

### 3.2. Photo Inhibitory Effect on Lesion Fluorescence Loss (∆F), Depth (L), and Integrated Mineral Loss (∆Z)

**Figure 3:**
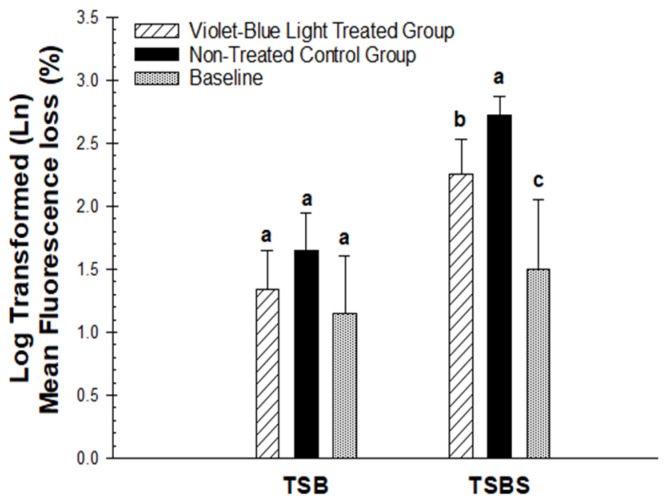
Comparison of mean fluorescence loss (∆F) in the baseline, violet-blue light treated, and non-treated groups of TSB and TSBS. Different letters indicate statistically significant differences between groups within TSB and TSBS, respectively.

The negative signs from the original data were removed from the parameters of QLF, such as ∆F, ∆Fmax, and ∆Q measurements, before the statistical analysis, so that larger values indicate larger lesions. Logarithmic transformations were done for the analysis. The QLF-D parameters of the violet-blue light-treated groups and non-treated group were not significantly different for TSB (*p* = 0.20 for ∆F, *p* = 0.20 for ∆Fmax, *p* = 0.17 for ∆Q, *p* = 0.18 for A_∆F_). There was a numerical decrease in the loss of fluorescence in the treated group, however, this was not statistically significant compared with the non-treated group (Figure 3).

The fluorescence radiance loss in the violet-blue light-treated group with TSBS was significantly less demineralized compared with the non-treated group for TSBS (*p* = 0.0116 for ∆F, *p* = 0.0168 for ∆Fmax, *p* = 0.0152 for ∆Q, *p* = 0.0224 for A_∆F_; Figure 3). The light-treated TSB group exhibited significantly less fluorescence loss compared with the treated TSBS (*p* = 0.0148 for ∆F, *p* = 0.0091 for ∆Fmax, *p* = 0.0025 for ∆Q, *p* = 0.0015 for A_∆F_) and non-treated groups (*p* = 0.0021 for ∆F, *p* = 0.0015 for ∆Fmax, *p* = 0.0002 for ∆Q, *p* = 0.0001 for A_∆F_; Figure 3). The comparison of mean fluorescence loss (∆F) imaged through QLF-D did not reveal any statistically significant differences between TSB and TSBS groups for the baseline (*p* = 0.55 for ∆F). However, the violet-blue light-treated group was significantly different from the baseline for TSBS (*p* = 0.0417) and the non-treated groups were significantly different from the baseline for TSBS (*p* = 0.0005). The baseline for TSB did not significantly vary from the violet-blue light treated (*p* = 0.64 for ∆F) and non-treated groups (*p* = 0.23 for ∆F) (Figure 3).

**Figure 4:**
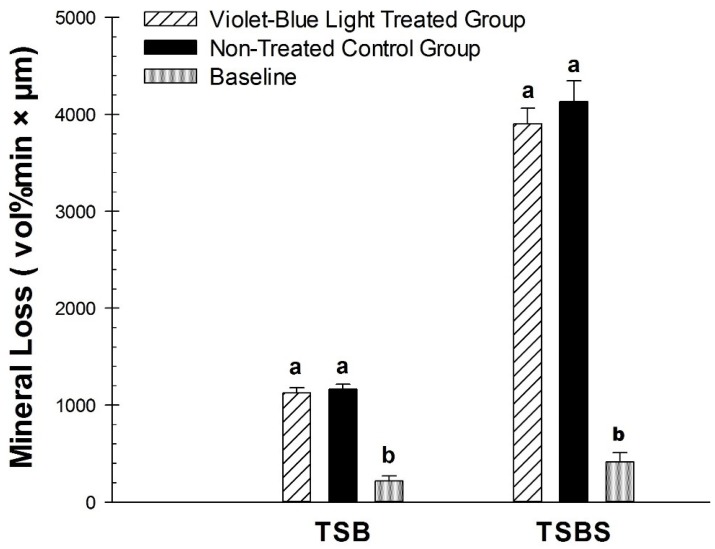
Comparison of mineral loss (∆Z) in the baseline, violet-blue light treated, and non-treated groups of TSB and TSBS. Treated and non-treated were significantly different for TSB. Comparison of lesion depth (L) in the baseline, violet-blue light treated, and non-treated groups of TSB and TSBS. Different letters represent significant differences between groups within TSB and TSBS, respectively.

**Figure 5:**
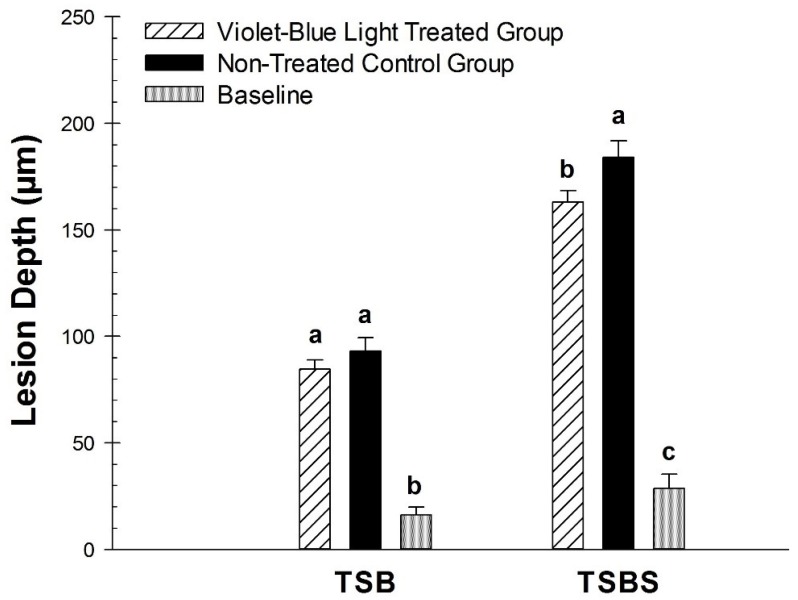
Comparison of lesion depth (L) in the baseline, violet-blue light treated, and non-treated groups of TSB and TSBS. Different letters represent significant differences between groups within TSB and TSBS, respectively.

There was a statistically significant difference for the violet-blue light treated group compared with the non-treated control group for ∆F on the dentin specimens incubated with *S. mutans* biofilm with TSBS (*p* < 0.02) (Figure 3). However, there was no significant difference observed for TSB (*p* = 0.24). Violet-blue light treated groups and non-treated control groups did not have significantly different mineral loss with TSB (*p* = 0.59) or TSBS (*p* = 0.36) (Figure 4). There were significant differences in the baseline values of lesion depth and mineral loss in the TSB and TSBS groups compared with the violet-blue light treated and non-treated groups (*p* < 0.0001) (Figure 4 and Figure 5).

## 4. Discussion

The purpose of this phototherapy study was to determine the effectiveness of violet-blue light in reducing the formation of lesions in dentin induced by *S. mutans*. Our biofilm model with two feedings of fresh 1% sucrose daily for 5 d develops a pro-cariogenic environment. *S. mutans* has a greater adherence to dentin through collagen binding proteins than enamel [36]. Violet-blue light with nine total treatments on *S. mutans* biofilm reduced bacterial numbers in the treated TSB and TSBS groups. A photo inhibitory effect with demineralization related to fluorescence radiance and lesion depth was seen with TSBS.

It is a well-known phenomenon that the number of TSBS-grown *S. mutans* biofilm cells is reduced as a result of an increase in the amount of extracellular polysaccharides (EPS), while the overall *S. mutans* biofilm mass increases in the presence of sucrose [37,38]. Lactic acid released from the metabolism of the biofilm cells on EPS is primarily responsible for caries formation. The effect of violet-blue light on EPS or glucans was not determined in this study. However, it has been shown previously that the biofilm architecture is affected by blue light and that the light reduces the amount of insoluble EPS [14,39]. The significant reduction of bacteria with violet-blue light on the sucrose-grown groups indicates the need to study in detail the photoinhibitory effect on glucans and pH variability. Increased bacterial numbers in TSB-grown cultures in comparison with TSBS-grown cells suggest that *S. mutans* with less EPS (i.e., TSB-grown *S. mutans*) has a greater ability to multiply rapidly rather than metabolizing sucrose [40]. A direct effect of violet-blue light on the bacteria as well as the EPS is important to prevent the progression of caries.

The loss of fluorescence related to integrated mineral loss measured with QLF was not significantly different between the treated and non-treated groups without sucrose. There was a numerical decrease in ∆F, ∆Q, and A_∆F_ in the violet-blue light group compared with the non-treated groups; however, the differences were not statistically significant with TSB. A short biofilm model for five days with minimal demineralization or the variability in A_∆F_ and ∆Q without sucrose could have contributed to the non-significance. In contrast, the ∆F for the violet-blue light-treated TSBS groups was significantly different from that of the non-treated groups.

The concept of photoinactivation with QLF was based on an observation of orange to red fluorescence in carious lesions in QLF images [41], probably owing to bacterial metabolic byproducts called porphyrins [30,42,43]. Porphyrins are fluorescent compounds or fluorophores produced by several bacteria [44]. Violet-blue light is absorbed by these fluorophores and the fluorophore undergoes a transition of energy of photons from a lower level to an excited level. At the excited state, the fluorophores react with the available molecular oxygen releasing free oxygen radical species that destroy proteins, lipids, and biological materials [5,45]. QLF works on a principle capturing the enamel surface of the tooth based on the underlying fluorescence from the dentin for any defects or lesions [46,47]. Few studies show the cutoff level with histological validation with natural dentin lesions including enamel [48,49,50,51]; however, this study was done exclusively on processed dentin specimens. Our study was not a validation study and does not provide any diagnostic values on sensitivity and specificity diagnostic values.

Violet-light on *S. mutans* biofilm at its most favorable growing conditions with adherence and accumulation on dentin specimens was studied to determine the actual effect of light in the most virulent conditions. The limitations of this study include the absence of in vivo conditions with saliva and pellicle formation on the substrate. Future studies should be conducted with multispecies biofilm with saliva and pellicle formation to mimic clinical conditions. *S. mutans* has an increased binding capacity with dentinal collagen compared twithenamel and with TSBS had reduced lesion depth in the violet-treated group. These results provide additional evidence for the effectiveness of violet-blue light as an adjunct prophylactic treatment to manage oral biofilm formation causing dentinal lesions.

The present study has shown that violet-blue light has photoinhibitory activity on *S. mutans* in minimizing the formation of biofilm on dentin specimens. According to our knowledge, there have been no studies reported similar to ours exclusively irradiating a *S. mutans* biofilm grown on dentin specimens. This in vitro study demonstrates a reduction in the number of bacteria that are necessary for the formation of carious lesions. Violet-blue light reduces bacterial numbers, thereby preventing accumulation of oral biofilm, and may contribute to a reduction of the formation of carious lesions. More in situ and clinical studies are needed to explore the effect of violet-blue light on dental plaque on tooth surfaces with and without the use of photosensitizers or reactive oxygen species producing substances.
